# The evolution of *Bordetella pertussis* has selected for mutations of *acr* that lead to sensitivity to hydrophobic molecules and fatty acids

**DOI:** 10.1080/22221751.2019.1601502

**Published:** 2019-04-10

**Authors:** Iain MacArthur, Thomas Belcher, Jerry D. King, Vasantha Ramasamy, Munirah Alhammadi, Andrew Preston

**Affiliations:** a The Milner Centre for Evolution, University of Bath, Bath, UK; b Department of Biology and Biochemistry, University of Bath, Bath, UK

**Keywords:** *Bordetella pertussis*, *acr*, evolution, growth inhibition, palmitate, repressor

## Abstract

Whooping cough, or pertussis, is resurgent in numerous countries worldwide. This has renewed interest in *Bordetella pertussis* biology and vaccinology. The *in vitro* growth of *B. pertussis* has been a source of difficulty, both for the study of the organism and the production of pertussis vaccines. It is inhibited by fatty acids and other hydrophobic molecules. The AcrAB efflux system is present in many different bacteria and in combination with an outer membrane factor exports acriflavine and other small hydrophobic molecules from the cell. Here, we identify that the speciation of *B. pertussis* has selected for an Acr system that is naturally mutated and displays reduced activity compared to *B. bronchiseptica*, in which the system appears intact. Replacement of the *B. pertussis* locus with that of *B. bronchiseptica* conferred higher levels of resistance to growth inhibition by acriflavine and fatty acids. In addition, we identified that the transcription of the locus is repressed by a LysR-type transcriptional regulator. Palmitate de-represses the expression of the *acr* locus, dependent on the LysR regulator, strongly suggesting that it is a transcriptional repressor that is regulated by palmitate. It is intriguing that the speciation of *B. pertussis* has selected for a reduction in activity of the Acr efflux system that typically is regarded as protective to bacteria.

## Introduction

The genus *Bordetella* consists of nine characterized species (recently other novel species have been reported), including those that cause respiratory infections. *B. pertussis* is a fastidious, Gram-negative coccobacillus that is a strict pathogen of humans that causes whooping cough or pertussis. *B. pertussis* has evolved recently from *B. bronchiseptica* or a *B. bronchiseptica-*like ancestor [1]. This speciation involved considerable genome reduction and rearrangement that resulted in an organism restricted to the human respiratory tract. In comparison, *B. bronchiseptica* has a broad host range and grows freely in the environment [2,3]*.* Pertussis is considered resurgent in many parts of the world, despite high levels of vaccination. Possible reasons for this have been well discussed (reviewed in [4,^5^,^6^–7]). Resurgence has emphasized sizeable gaps in the understanding of the mechanisms of *B. pertussis* pathogenesis and of *B. pertussis* physiology. *In vitro*, growth of *B. pertussis* has proved challenging, especially in liquid culture. *B. pertussis* is unable to metabolize sugars, appearing reliant on the metabolism of amino acids [8,9]. Glutamate and proline are the most readily oxidized and glutamate has been used as the primary carbon and nitrogen source in most defined *B. pertussis* growth media, for example Stainer–Scholte (SS) broth [9]. *B. pertussis* is unable to grow in some media, even though they contain sources of glutamate, for example on Luria–Bertani agar, due to the presence of compounds inhibitory to growth. *B. pertussis* is sensitive to a number of compounds including peptone, fatty acids, sulphur, peroxide, and manganese [10]. Often, charcoal, blood, or cyclodextrins are added to *B. pertussis* growth media to sequester hydrophobic inhibitory compounds. In particular, growth of *B. pertussis* is inhibited by a number of fatty acids including palmitic acid (16:0) [10]. However, in the commonly used laboratory media *B. pertussis* releases fatty acids, particularly palmitic acid, into the culture supernatant to concentrations that inhibit growth [11]. This contributes to a poor yield from *in vitro B. pertussis* cultures and to issues with reproducibility of the quality of the biomass produced, creating major problems for vaccine manufacturers. This will be particularly challenging for increasing vaccine production as part of any intervention to combat resurgence.

Many bacteria possess mechanisms to resist the inhibitory effects of small hydrophobic molecules, for example the AcrAB-TolC efflux system of *Escherichia coli* that is responsible for resistance to a wide range of hydrophobic inhibitors [12]. This system consists of an inner membrane transporter AcrA, a periplasmic coupling protein, AcrB, that couples AcrA to TolC, the outer membrane channel [13,14]. In addition, *E. coli* AcrZ associates with AcrB to enhance the export of some substrates [15]. Here, we report that the *B. pertussis acrAB-cusC* (*acrABC*) locus contains two small deletions, resulting in low activity of *B. pertussis* AcrABC compared to the *B. bronchiseptica* system. We demonstrate that *Bordetella* AcrABC confers resistance to inhibition of growth by acriflavine and fatty acids and that a LysR-type transcriptional regulator represses transcription of *acrABC*, repression that is alleviated by the substrate palmitate.

## Materials and methods

### Bacterial strains and culture conditions

Bacterial strains used in this study are listed in Table 1. Plasmids used in this study are listed in Table 2. BP536 is a single passage, streptomycin resistant derivative of Tohama I and used as WT in this study. *B. pertussis* was cultured on charcoal agar (CA) (Fisher Scientific, Loughborough, UK) for 3 days at 37°C or in Stainer–Scholte broth (SS) or in SSH (SS with 1 g/L heptakis [9] (Sigma-Aldrich, Gillingham, UK)) at 37°C with shaking at 180 rpm. *E. coli* were grown on LB agar or in LB broth at 37°C. Antibiotics were used where appropriate at the following concentrations: kanamycin 50 µg/ml and gentamycin 30 µg/ml. For growth of *E. coli* ST18 [16] media was supplemented with amino-levulinic acid at 50 µg/ml.

**Table 1.** Strains used in this study.

Strain	Description	Reference
*B. pertussis* BP536	WT	[30]
*B. bronchiseptica* RB50	WT	[31]
*E. coli* ST18	S17 λ*pir* Δ*hemA*	[16]
*E. coli* NEB5α	Cloning strain	NEB
BPΔ*0983*	Deletion of BP0983	This study
BPΔ*acrABC*	Deletion of *acrA-C*	This study
BP*acrABC*_BB_	Allelic replacement of BP*acrABC* with *B. bronchiseptica acrABC*	This study
BP*acrABC*_BB_Δ*0983*	Deletion of BP*0983* in BP*acrABC*_BB_	This study
BPpBBRK	Wild-type containing pBBRK	This study
BP*acrABC*_BB_pBBRK	Wild-type containing pBBRK*acrABC*_BB_	This study

**Table 2.** Plasmids used in this study.

Name	Description	Source or reference
pSS4940	pSS4245-based suicide plasmid (Gm^R^)	[32]
pSS4940GG	pSS4245-based suicide plasmid modified for Golden Gate cloning (Gm^R^, Cm^R^)	This study
pBBRK	pBBR-based shuttle vector, Kan^R^	[33]
pBBRKGG	pBBRK shuttle vector modified for Golden Gate cloning (Kan^R^, Cm^R^)	This study
pSS4940*0983*AB	pSS4940 containing upstream and downstream fragments of BP*0983* for allelic exchange	This study
pBBRK*acrABC*_BB_	pBBRK containing BB*2526*-*acrABC*_BB_	This study
pSS4940acrDelK	pSS4940 containing 5’ fragment of *acrA*, Kan^R^ cassette and 3’ fragment of *acrC*	This study
pSS4940*acrABC*_BB_	pSS4940 containing BB*2526*-*acrABC*	This study

### PCR

PCR was conducted using OneTaq 2x master mix (NEB, Hitchin, UK) following the manufacturer's instructions modified to include 0.75 µl of DMSO per reaction. The cycle conditions were as follows: 94°C for 4 min followed by 30 cycles of 94°C for 30 s, annealing for 1 min, 68°C for 1 min/kb and 5 min at 68°C. Details for the primers used can be found in Table 3.

**Table 3.** Primers used in this study.

Primer	Sequence 5’–3’	Annealing temp (°C)	Comment
0983AF	AAAAGGTCTCTCGAGAGAACAGGCGCTTCTGGAC	52	*BP0983* mutagenesis
0983AR	AAAAGGTCTCGATGTGGAGTTCCATGCCTTGCAAAC	52	*BP0983* mutagenesis
0983BF	AAAAGGTCTCTACATAACACTGGCAGGCCAGAATAA	54	*BP0983* mutagenesis
0983BR	AAAAGGTCTCGAACTACTGGTCCTCACGCAGGAT	54	*BP0983* mutagenesis
acrDelAF	AAAAAAGGTCTCTCTAGAGCAAACGGTTCATGGCGGATG	58	*acr*_BP_ deletion
acrDelAR	AAAAAAAGGTCTCCATATGAGCAAGGTGGCGGTCTTCA	58	*acr*_BP_ deletion
acrDelBF	AAAAAAGGTCTCAGATCTACAACGCCTACCTGACCCTG	58	*acr*_BP_ deletion
acrDel BR	AAAAAAGGTCTCGAATTCGCGATGCCGATATCCTTCTG	58	*acr*_BP_ deletion
KanF	AAAAAAGGTCTCCATATGACGTCTTGTGTCTCAAAATCTC	45	Amplification of Kan^R^
KanR	AAAAAAGGTCTCAGATCTTAGAAAAATTCAATCCAGCATC	45	Amplification of Kan^R^
BB2526/acrABCF	ACAAAAAAGCAGGCTCCGAATTCTTAACGCAAATTTAAAAACGCC	53	Amplification of *BB2526*/*acrABC*_BB_
BB2526/acrABCFR	GGGACAACAAGGTTCTCATGACGGCAGGT	53	Amplification of *BB2526*/*acrABC*_BB_
BBcusCF	CATGAGAACCTTGTTGTCCCTTG	53	Amplification of *acrC*_BB_
BBcusCR	CAAGAAAGCTGGGTCGAATTTCATCGTGTTGTTCGTCGT	53	Amplification of *acrC*_BB_
acrAq1F	CTTTCCTTGGCGGCATTGAC	60	qPCR of *acrA*
acrAq1R	CGACTGGGTTTTCAGGGTGA	60	qPCR of *acrA*
recAqF	AACCAGATCCGCATGAAGAT	60	qPCR of *recA*
recAqR	ACCTTGTTCTTGACCACCTT	60	qPCR of *recA*
0983qF	CCTGGCCATTCCCAAATCCA	60	qPCR of BP*0983*
0983qR	TGGCAGCGCTCGAAGTAAAG	60	qPCR of BP*0983*

### Mutagenesis

Deletions of *acrABC* and BP*0983* were created by amplifying by PCR approximately 500 bp of the regions flanking the deletions. For *acrABC*, these flanking regions were cloned either side of a kanamycin resistance cassette to produce acrDELKanABpSS4940. For deletion of BP0983, the flanking regions were joined together to create an in-frame deletion of *BP0983.* PCR fragments were cloned into pSS4940GG by Golden Gate cloning [17]. Constructs were transformed into chemically competent *E. coli* ST18, which was subsequently used as the donor for conjugation. Conjugations were carried out as previously described [18]. Conjugants were selected on charcoal agar supplemented with gentamycin and 50 mM MgSO_4_. Following counter-selection against merodiploids, deletion mutants were distinguished from WT by PCR. For replacement of the *B. pertussis acr* locus with that of *B. bronchiseptica*, *B. bronchiseptica* locus was amplified by PCR as two sections and ligated together using Gibson Assembly. The resulting locus fragment was cloned into pSS4940GG as above and conjugated into BPΔ*acrABC*.

### Inhibition assays

Plate grown *B. pertussis* strains were resuspended in SS to an OD_600_ of 0.1 and grown overnight. Cultures were diluted in fresh SS supplemented with different concentrations of the inhibitory compound: acriflavine, palmitic acid, myristic acid, oleic acid, and decanoic acid (Sigma-Aldrich, BioXtra grade). Cultures (200 μl) were incubated at 37°C with shaking at 200 rpm in microtitre plates. After 3 days, the OD_600_ was measured in a Fluostar Omega plate reader (BMG LabTech, Aylesbury, UK). The OD_600_ of cultures grown in the presence of inhibitor was divided by the OD_600_ of the same strain grown without inhibitor to give relative growth as a percentage. Resistance to ampicillin was determined using E-test strips in accordance with manufacturer's instructions (Biomerieux, Basingstoke, UK). Plates were inoculated with 100 µl of a suspension of bacteria at OD_600_ = 0.8 (∼1.5 × 10^8^ cfu/ml).

### RT-qPCR

Bacteria were grown overnight in SS or SSH and these cultures were used to inoculate fresh media at an OD_600_ of 0.05. Cultures were harvested at OD_600_ = 0.9 ± 0.1 by centrifugation (4000*g* for 10 min) and resuspended in 700 µl of Tri-reagent (Invitrogen; ThermoFisher, Loughborough, UK), vortexed vigorously, and frozen at −80°C. Nucleic acids were precipitated with ethanol, DNA was removed using 4U of Turbo DNase (Ambion, ThermoFisher) for 1 h, and RNA was purified using the RNeasy kit (Qiagen, Manchester, UK) in accordance with the manufacturer's instructions. The concentration of RNA was determined using Qubit broad range RNA quantification kit (Fisher Scientific). RNA integrity was determined by agarose gel electrophoresis. Finally, RNA was confirmed as being DNA free by PCR using 50 ng of RNA as template in PCR with *recA*F and *recA*R primers. First strand cDNA was synthesized using ProtoScript II (NEB) with 1 µg of total RNA as template and 6 µM random primers and incubated for 5 min at 25°C, 1 h at 42°C. The reaction was stopped by incubating at 65°C for 20 min. cDNA was diluted 1/30 in H_2_O for use in qPCR.

qPCR was run on an a StepOne Real-time PCR System (Applied Biosystems, ThermoFisher) using SyberGreen Turbo Master mix (Applied Biosystems), in a total reaction volume of 25 µl with primers at 300 nM. Triplicate reactions were run for each sample. Reactions conditions were: 95°C for 10 min and 40 cycles of 95°C for 15 s and 1 min at 60°C. The housekeeping gene *recA* was used to calculate ΔCT and ΔΔCT was determined by determining the difference between the reference condition and experimental condition. Relative expression was represented as fold change (fold change = 2^−ΔΔCT^). Three biological repeats were used for each experiment. Significance was determined using Students *t*-test using a hypothetical value of 1 (no relative expression difference).

### Ethidium bromide accumulation assay [19,20]

*B. pertussis* strains were grown in SS until OD_600_ = 1.00 ± 0.1. Ethidium bromide was added at 16 µM. Samples were vortexed and transferred to a 96-well plate, stored in the dark for 30 min before fluorescence was measured in a FLOUstar Optima microplate reader (BMG Labtech). Variation in OD_600_ was accounted for by dividing the fluorescence value by the OD_600_ and expressed as relative fluorescence.

### Modelling the structure of BP0983

This was conducted using I-TASSER under default settings [21]. The C-score of the model produced by I-TASSER was determined by comparison to a number of similar protein sequences the most similar to BP0983 was CrgA Protein Data Bank number 3hhgE [22]. Model images were made using CHIMERA 1.13.1 [23].

## Results

### Deletions in *acrA* and *cusC* in *B. pertussis* results in inhibition of growth on LB agar

**Figure 1.**
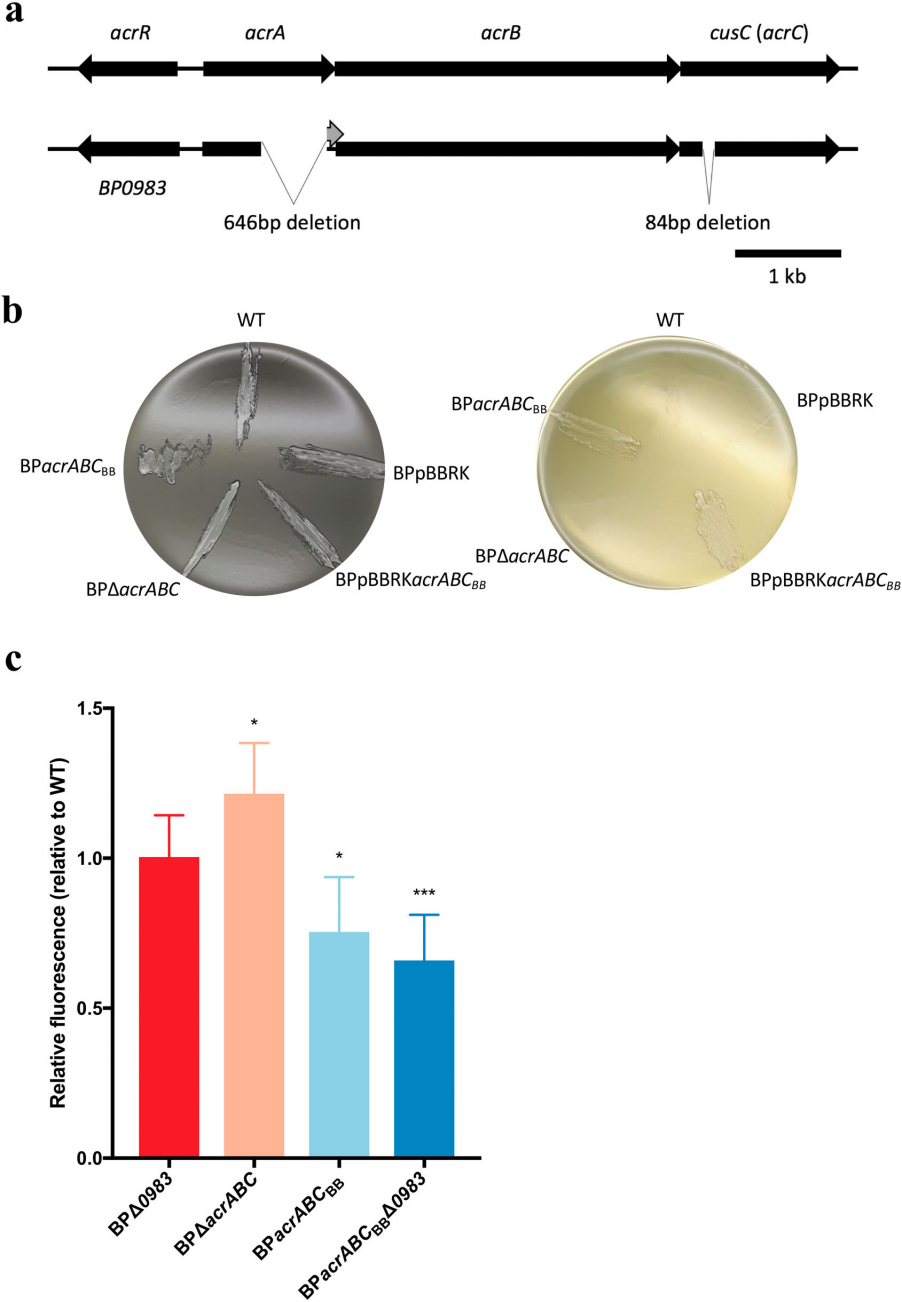
*B. pertussis acr* is mutated and has lower activity than *B. bronchiseptica acr*. (a) Diagram showing the arrangement of the *B. pertussis* and *B. bronchiseptica acrABC* loci. BP0983/BB2526 encodes a LysR-type transcriptional regulator. *B. pertussis acrA* and *acrC* (*cusC*) have 646 and 84 bp (in-frame) deletions respectively, compared to *B. bronchiseptica*. (b) *B. pertussis* strains were plated on charcoal agar (left) and LB agar (right). All strains grew on charcoal agar. WT, WT containing pBBRK (BPpBBRK) and BPΔ*acrABC* were unable to grow on LB agar whereas the presence of *acrABC*_BB_ conferred growth on LB. (c) An ethidium bromide accumulation assay was used to measure AcrABC activity. *acrABC_BB_* conferred greater efflux activity compared to that attributable to the *B. pertussis* locus. Deletion of *acrABC* from *B. pertussis* resulted in decreased activity suggesting *B. pertussis* AcrABC has residual function. The data is based on six independent experiments. Error bars represent standard deviation and significance was determined by a one-sample *t*-test comparing each strain to WT. *: *p* < 0.05; **: *p* < 0.01.

*B. pertussis* is sensitive to free fatty acids and other hydrophobic molecules, and its growth is limited on rich media including Luria–Bertani media [24], whereas *B. bronchiseptica* is less sensitive. During comparisons of genome sequences of *B. pertussis* Tohama I and *B. bronchiseptica* RB50, it was noticed that while both species contain homologues of *acrAB-tolC* genes, the *B. pertussis* system contains two mutations. Compared to *B. bronchiseptica*, *B. pertussis acrA* (BP0984) contains a 646 bp deletion of the 3’ region of the gene, predicted to result in a truncated AcrA that due to a frameshift created by the deletion contains different carboxyl-terminal amino acids to that of *B. bronchiseptica* AcrA. Also, there is an 84 bp in-frame deletion in *BP0986,* originally annotated as *cusC*, compared to the homologue in *B. bronchiseptica*, which we predict to encode the outer membrane factor of the efflux system, and refer to here as *acrC* (Figure 1a). Aside from these two deletions, the two loci are highly homologous. There are just five amino acid differences between the two AcrB proteins (1059 amino acids) and (outside of the 28 amino acid deletion) one amino acid difference between the two AcrC proteins. Furthermore, these deletions are conserved across all currently available *B. pertussis* genome sequences, indicating that these deletions were likely acquired early in the evolution of *B. pertussis*. We hypothesized that *B. pertussis* contains a non-functional Acr efflux system whereas it is functional in *B. bronchiseptica*. To test this, the *B. bronchiseptica* locus (*BB2527-9)*, including the adjacent gene encoding a putative LysR-type transcriptional regulator (*BB2526*) carried on pBBRK, a plasmid that replicates in *Bordetella*, was introduced into *B. pertussis* to produce BPpBBRK*acrABC*_BB_. Also, *acrA-C* were deleted from *B. pertussis* and replaced with a kanamycin resistance cassette to produce BPΔ*acrABC.* This strain was used as a background to clone the *B. bronchiseptica* locus onto the chromosome in place of the kanamycin cassette, effectively replacing the *B. pertussis acr* locus with that of *B. bronchiseptica*, producing BP*acrABC*_BB_.

The ability of each strain to grow on LB agar was assessed. WT *B. pertussis*, WT containing pBBRK plasmid alone, and BPΔ*acrABC* were unable to grow on LB agar, in contrast to both BPpBBRK*acrABC*_BB_ and BP*acrABC*_BB_ (Figure 1b), suggesting that the inability of *B. pertussis* to grow on LB is caused by the deletions in BP*acrA* and BP*acrC*.

### *B. pertussis* AcrABC retains residual function

The relative activities of the *B. pertussis* and *B. bronchiseptica* Acr systems were investigated by assaying the accumulation of ethidium bromide within strains, that in other bacteria is effluxed by Acr [19,20]. High levels of bacterial fluorescence were indictive of a low level of efflux of ethidium bromide, and this was represented relative to WT to control for variation in fluorescence between experiments (Figure 1c). BPΔ*acrABC* accumulated more ethidium bromide than WT (22% greater than WT, *p* = 0.026), indicating that the deletion of *acrABC* decreased efflux of ethidium bromide. This suggested that *B. pertussis* AcrABC retains some function, despite the two mutations. The presence of the *B. bronchiseptica* locus resulted in significantly less accumulation of ethidium bromide (24% less than WT, *p* = 0.021) demonstrating that the *B. bronchiseptica* system has greater activity for efflux of ethidium bromide than that of *B. pertussis*.

### BP0983 is a transcriptional repressor of *acrABC*

**Figure 2.**
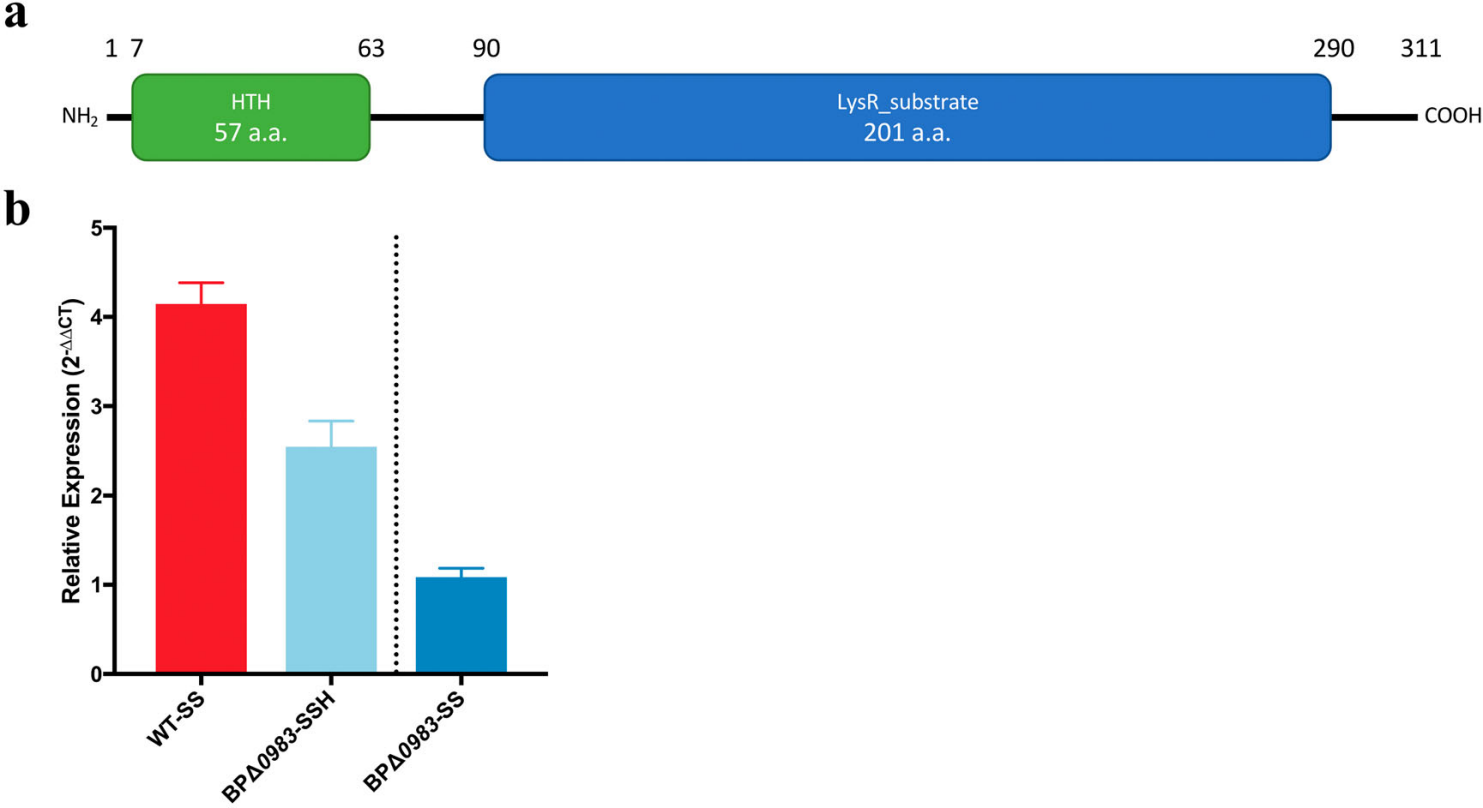
BP0983 is a transcriptional repressor of *acrABC*. (a) Diagram of the conserved domains of the LysR-family transcriptional regulator encoded by *BP0983*. A predicted helix-turn-helix motif (green) at the N-terminus is indicative of repressor activity. It also contains a LysR substrate domain (blue). (b) Expression of *acrA* determined by RT-qPCR. Growth of WT in SS broth with heptakis (SSH) was the reference condition for comparison on the left of the dotted line, and BPΔ*0983* in SSH on the right. There was a significant increase in expression of *acrA* in WT in SS broth without heptakis (WT-SS) and BPΔ*0983* SSH (BPΔ*0983*-SSH) with a fold change of 4.1 and 2.5 respectively. There was no difference between *acrA* expression in BPΔ*0983* in SS compared to SSH. The data is based on three biological repeats. Error bars represent standard deviation and significance was determined by one-sample *t*-test comparing each condition to the reference.

Adjacent to, but divergent from, *acrABC* in both *B. pertussis* and *B. bronchiseptica* is a gene encoding a putative LysR family transcriptional regulator (BP0983 and BB2526 respectively, Figure 1). Genes encoding this class of regulator are often situated upstream of, and divergent from, the genes that they regulate [25]. The predicted structure of BP0983 contains two conserved domains, an N-terminal helix-turn-helix (HTH) domain found in LysR family proteins (pfam00126) and a LysR substrate binding domain (pfam03466) (Figure 2a). An N-terminal HTH is the characteristic of LysR-type transcriptional regulators that act as transcriptional repressors.

To test the involvement of BP0983 in regulating *acr* expression, *BP0983* was deleted from *B. pertussis* in both the WT and BP*acrABC*_BB_ backgrounds, producing BPΔ*0983* and BP*acrABC*_BB_Δ0983 respectively. The relative level of transcription of *acrA* in WT and BPΔ*0983* was measured using RT-qPCR. Strains were grown in SS broth either with or without heptakis. Heptakis is a cyclodextrin used to supplement *B. pertussis* growth media by sequestering small hydrophobic molecules that are inhibitory to *B. pertussis* growth. WT grown with heptakis was used as the reference condition. Compared to this, there was a 4.1-fold increase in the expression of *acrA* in WT when grown in SS without heptakis (Figure 2b). *B. pertussis* growing in SS broth produces free fatty acids that are sequestered by heptakis when present in the medium [11]. The increased transcription of *acrA* in the absence of heptakis, and thus in the presence of free fatty acids in the culture medium, is consistent with the notion that free fatty acids signal to increase transcription of *acr*. In BPΔ*0983*, transcription of *acrA* was increased, and this was insensitive to the presence or absence of heptakis (2.5-fold with heptakis and 2.8-fold without heptakis, relative to WT).

Deletion of BP*0983* resulted in increased levels of transcription of *acrA*, but regardless of the presence or absence of heptakis, and thus of the presence of fatty acids in the culture medium. This supports the idea that BP0983 is a transcriptional repressor of *acrABC* and that fatty acids derepress transcription. However, deletion of BP*0983* would be expected to completely relieve repression of *acrA* transcription and it is not clear why the level of expression of *acrA* in BPΔ*0983* was not as high as in WT grown in SSH, suggesting that there may be a synergistic repressor activity by something that is sequestered by heptakis.

### The role of Acr in resistance to acriflavine, ampicillin and fatty acids

*E. coli* AcrAB-TolC expels acriflavine and other small hydrophobic molecules from the cell. To test the activity of *Bordetella* Acr against such compounds, the sensitivity of strains to acriflavine, ampicillin and fatty acids was tested. BPΔ*acrABC* proved difficult to grow in broth in the absence of heptakis. The inability to grow this strain in the absence of heptakis, that might sequester the test compounds, precluded it from being included in these assays.

**Figure 3.**
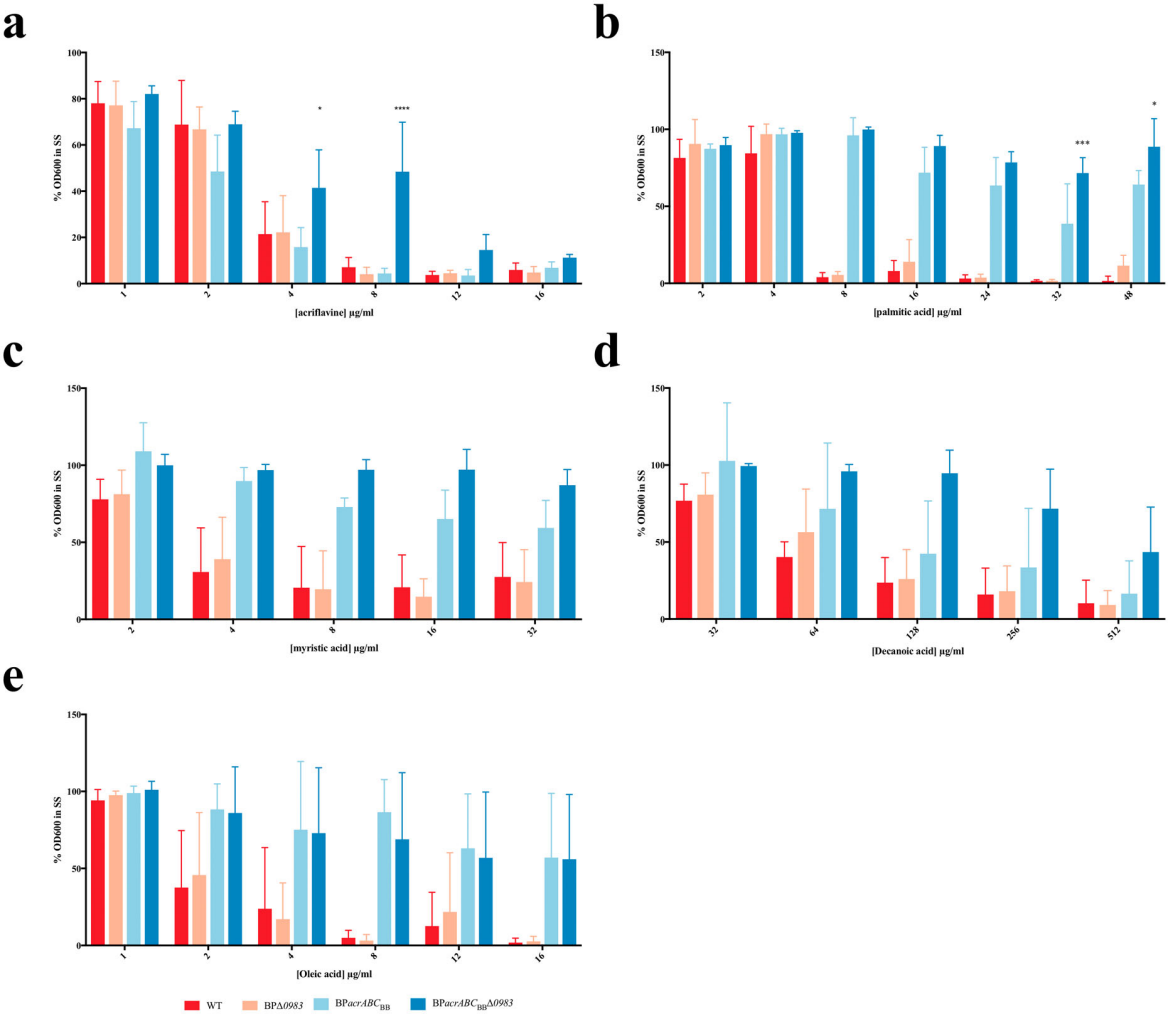
The role of Acr in resistance to inhibition of growth by small hydrophobic molecules. *B. pertussis* WT (red), BPΔ*0983* (orange), BP*acrABC*_BB_ (light blue), and BP*acrABC*_BB_Δ*0983* (dark blue) were grown in SS broth overnight and seeded into 96-well plates with various concentrations of acriflavine (a), palmitate (b), myristate (c), dodecanoate (d), and oleate (e). Plates were incubated at 37°C for 48 h after which OD_600_ was measured. The OD_600_ for each strain was represented as a percent of the OD_600_ of untreated samples. Only BP*acrABC*_BB_Δ*0983* was resistant to acriflavine at ≥8 µg/ml. BP*acrABC*_BB_ and BP*acrABC*_BB_Δ*0983* were resistant to higher levels of fatty acids than either WT or BPΔ*0983*. The data is based on three independent experiments. Error bars represent standard deviation and significance was determined by 2-way ANOVA.

The growth of strains over 48 h in the presence of different concentrations of acriflavine was measured and normalized to growth without acriflavine. There was no significant difference between growth of any of the strains at ≤4 µg/ml acriflavine. However, at 8 µg/ml the growth of BP*acrABC*_BB_Δ*0983* was significantly greater than any of the other strains (Figure 3a). These data suggest that *B. pertussis* AcrABC could efflux acriflavine, but with low activity as growth of BPΔ*0983*, in which the expression of the system was higher, was no different to WT. The increased growth of BP*acrABC*_BB_ suggests that the *B. bronchiseptica* locus conferred increased resistance to acriflavine but only when repression of its transcription was relieved by deletion of *BP0983*. In turn, this suggested that although acriflavine was a substrate of Acr, it was unable to induce relief of BP0983-mediated repression.

The role of Acr in resistance to ampicillin was tested using E-test strips. The MIC for WT was 0.094 µg/ml. The MIC for BPΔ*acrABC* (0.19 µg/ml) and for BPΔ*0983* (0.25 µg/ml) was not largely different to that of WT. The MICs of BP*acrABC*_BB_ (16 µg/ml) and BP*acrABC*_BB_Δ*0983* (>256 µg/ml) suggested that the *B. bronchiseptica* locus conferred significantly increased resistance to ampicillin, and that the sensitivity of *B. pertussis* to ampicillin may, at least in part, be due to the low activity of *B. pertussis* Acr.

Palmitate is the main fatty acid released by *B. pertussis* during growth [11]. The role of Acr in the sensitivity of *B. pertussis* to palmitate was investigated (Figure 3b). There was no difference between the growth of strains in the presence of palmitate up to 4 µg/ml. Above this level, the growth of strains containing *acrABC*_BP_ was inhibited, even if transcription of it was derepressed (BPΔ*0983*). There was a significant difference in the growth of BP*acrABC*_BB_ in the presence of palmitate compared to both WT and BPΔ*0983*, demonstrating that AcrABC_BB_ conferred much higher levels of resistance to growth inhibition by palmitate than AcrABC_BP_ (Figure 5B). There was a significant difference in growth between BP*acrABC*_BB_ and BP*acrABC*_BB_Δ*0983* at both 32 and 48 µg/ml of palmitate (Figure 3b), consistent with the deletion of *BP0983* increasing the level of expression of *acrABC*_BB_.

To determine the range of fatty acids that are substrates for AcrABC, growth in the presence of decanoic acid (C10:0), myristic acid (C14:0), and oleic acid (C18:1) was tested (Figure 3c–e). There was a similar pattern of resistance to that observed with palmitate in that AcrABC_BB_ conferred higher levels of resistance than AcrABC_BP_ and deletion of *BP0983* increased further the resistance conferred by AcrABC_BB_, although this increase was not always significant. The data suggested that short chain fatty acids inhibited the growth of *B. pertussis* less than longer chain fatty acids.

### BP0983 contains a potential fatty acid binding site

**Figure 4.**
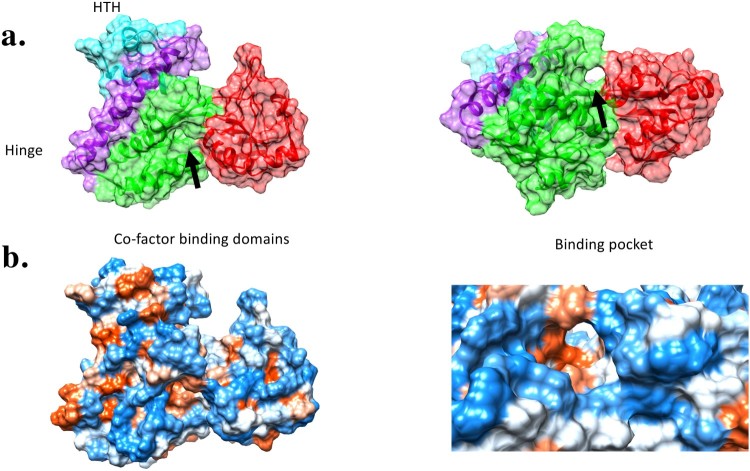
Modelling the structure of BP0983 identifies a putative hydrophobic channel. (a) A model of the structure of the LysR transcriptional regulator, BP0983, using I-Tasser (default settings), based on a number of similar proteins including CrgA (PDB: 3hhgE) [22], with a C-score of 0.65. The structure comprises a helix-turn-helix DNA binding domain (blue), a hinge domain (purple), and two co-factor binding domains (green and red). The LysR co-factor binding site is found between the two co-factor binding domains*.* (b) Mapping of hydrophobic amino acids (red) reveals that the co-factor binding region contains a channel lined by hydrophobic amino acids that could be compatible with binding fatty acids such as palmitate.

*Bordetella* AcrABC was active against a range of fatty acids, and *B. pertussis* releases palmitate during its growth *in vitro*. We hypothesized that fatty acids signalled to increase expression of *acrABC* by binding to BP0983 to relieve its repression of *acrABC.* A structural model of BP0983 was produced using I-TASSER [21] (Figure 4). The model had a high C-score (0.42) and a TM-score (0.77 ± 0.10), which was indicative of a realistic prediction of the structure of BP0983. This structure was consistent with that of LysR transcriptional regulators, comprising a DNA binding domain, a hinge domain that allows for a conformational change following the binding of a co-factor which interacts with a binding pocket that is formed between the two lobes of the co-factor binding domain. This putative binding pocket forms a channel lined with hydrophobic amino acids and this would appear to be compatible with the binding of fatty acids.

### Palmitate induces the expression of *acrA*

**Figure 5.**
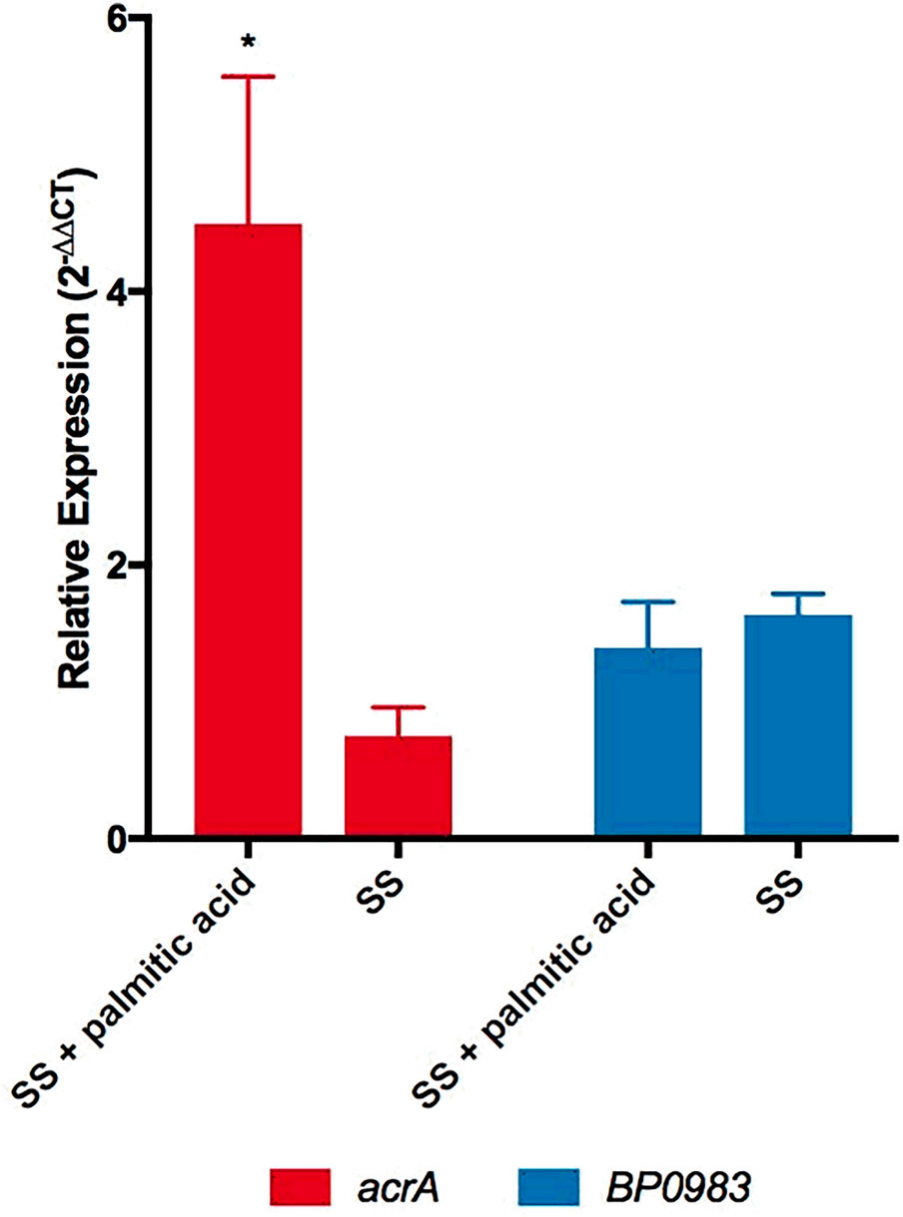
Palmitate induces the expression of *acrA*. Expression of *acrA* (red) and BP*0983* (blue) was measured by RT-qPCR. *B. pertussis* was grown overnight in SS broth with heptakis and then inoculated into fresh SS with or without 16 µg/ml palmitate. A sample was taken (*t* = 0) (reference condition) after 1 hour and RNA was extracted from these. The presence of palmitate induced a 4.5-fold increase in transcription of *acrA* (*p* = 0.0302). There was no increase in expression of *acrA* when incubated in SS alone. There was no increase in expression of BP0983 in either SS with palmitic acid or SS. The data is based on three biological repeats. Error bars represent standard deviation and significance was determined by one-sample *t*-test.

It was reasoned that if palmitate interacts with BP0983 to relieve repression of *acrABC* then adding palmitate to *B. pertussis* would be expected to increase the expression of the locus. To test this, the transcription of *acrA* was determined by RT-qPCR. Cultures of WT were grown in the presence of heptakis to sequester palmitate produced during growth that might derepress transcription of *acrA* in all samples. Bacteria were diluted into fresh SS broth or SS broth supplemented with palmitic acid (16 µg/ml) and after incubation for 1 h RNA was extracted for RT-qPCR analyses (Figure 5). Relative to the level of expression of the housekeeping gene, *recA*, the expression of *acrA* increased 4.5-fold in the presence of palmitate (*p* = 0.0302) (Figure 5). This is consistent with palmitate interacting with BP0983 to relieve repression of *acrABC* transcription. In the control culture without palmitate, the expression of *acrA* decreased slightly during the hour of incubation, suggesting that even in the presence of heptakis, in the original culture some free palmitate caused a slight derepression of transcription, but that diluting the bacteria into fresh SS broth in which there was no palmitate led to full repression and thus a decrease in transcription of *acrA.* There was no significant change in the expression of *BP0983* either in the presence or absence of palmitate (Figure 5), demonstrating that the change in transcription of *acrA* was not due to a change in the level of the repressor itself.

## Discussion

The role of AcrAB-TolC as an efflux pump of hydrophobic molecules with an emphasis on its role in antimicrobial resistance [26,^27^–28] has been described. This study reveals that *Bordetella* AcrABC is as a non-specific efflux pump of small hydrophobic molecules. Intriguingly, *B. pertussis* has suffered two deletions, in *acrA* and *cusC* (that we have referred to as *acrC*) that results in a reduction in activity of *B. pertussis* AcrABC leading to an increase in sensitivity to ampicillin, acriflavine and fatty acids. However, *B. pertussis* AcrABC retains low level function rather than being non-functional, as a strain from which the locus was deleted accumulated more ethidium bromide than WT, and in SS in the absence of heptakis this strain grew less than WT, consistent with increased sensitivity to inhibitory compounds present in the growth media. *B. bronchiseptica*-derived AcrABC demonstrated significantly greater activity than the *B. pertussis* system, revealing that it is active against acriflavine, ampicillin but particularly fatty acids. A transcriptional repressor controls the expression of *Bordetella acrABC* and we show data consistent with palmitate interacting with the repressor to control its activity, such that *B. pertussis* senses palmitate to increase the expression of an efflux pump that protects the cell from the inhibitory effect of the fatty acid. It is logical for the *Bordetella* to possess an efflux system that protects them from the effects of free fatty acids that they secrete during their growth, the reason for which is unknown. However, it is unclear why *B. pertussis* has evolved to possess a poorly functional AcrABC efflux system that appears to render the organism highly susceptible to the palmitate that it secretes. It is possible that palmitate secretion is an artefact of growth *in vitro*. Alternatively, *B. pertussis* may secrete palmitate but in its natural niche, the human respiratory tract, the palmitate does not build to levels that are inhibitory*.* In other bacteria, high levels of Acr expression can result in excretion of metabolites, resulting in growth inhibition of the organism [29]. Thus the low activity AcrABC_BP_ may provide sufficient protection from hydrophobic molecules within its niche, but prevent excretion of important metabolites. *B. parapertussis* is thought to have evolved, like *B. pertussis*, from a *B. bronchiseptica-*like ancestor to become a cause of whooping cough in people and restricted to the human respiratory tract as its sole niche, although there appears to be an ovine-associated lineage of *B. parapertussis* distinct to the human-associated strains. Examination of *B. parapertussis* genome sequences reveals that these bacteria contain the full-length *acrABC* locus that is over 99% identical to *acrABC_BB_* at the nucleotide level and thus, like *B. bronchiseptica*, contains a high-activity efflux system. Thus the evolution of a low-activity AcrABC appears to be specific to *B. pertussis* and its unique pathogenicity and understanding will contribute to deciphering this important host–pathogen interaction.
